# Recognizing Personality Traits Using Consumer Behavior Patterns in a Virtual Retail Store

**DOI:** 10.3389/fpsyg.2022.752073

**Published:** 2022-03-11

**Authors:** Jaikishan Khatri, Javier Marín-Morales, Masoud Moghaddasi, Jaime Guixeres, Irene Alice Chicchi Giglioli, Mariano Alcañiz

**Affiliations:** Instituto de Investigación e Innovación en Bioingeniería (i3B), Universitat Politécnica de Valencia, Valencia, Spain

**Keywords:** Big Five personality, consumer behavior, eye-tracking (ET), navigation, machine learning, statistical learning, virtual store, virtual reality

## Abstract

Virtual reality (VR) is a useful tool to study consumer behavior while they are immersed in a realistic scenario. Among several other factors, personality traits have been shown to have a substantial influence on purchasing behavior. The primary objective of this study was to classify consumers based on the Big Five personality domains using their behavior while performing different tasks in a virtual shop. The personality recognition was ascertained using behavioral measures received from VR hardware, including eye-tracking, navigation, posture and interaction. Responses from 60 participants were collected while performing free and directed search tasks in a virtual hypermarket. A set of behavioral features was processed, and the personality domains were recognized using a statistical supervised machine learning classifier algorithm *via* a support vector machine. The results suggest that the open-mindedness personality type can be classified using eye gaze patterns, while extraversion is related to posture and interactions. However, a combination of signals must be exhibited to detect conscientiousness and negative emotionality. The combination of all measures and tasks provides better classification accuracy for all personality domains. The study indicates that a consumer’s personality can be recognized using the behavioral sensors included in commercial VR devices during a purchase in a virtual retail store.

## Introduction

Due to technological advancements over the last two decades, virtual environments have thrived and elicited novel and exciting consumer experiences. This popularity has resulted in a surge of the use of such advancements in the commercial application of virtual reality (VR). Researchers are increasingly using VR to simulate natural phenomena and social interactions, creating interactive and multimodal sensory stimuli that offer unique advantages over other neuroscientific research methodologies (Bohil et al., 2011). Scientists have made VR technology compatible with measurement of human behavior, allowing for the presentation of multimodal stimuli with a high degree of ecological validity and control. It has been shown that the neural mechanisms that humans exhibit when immersed in virtual environments are very similar to those that emerge in real life (Alcañiz et al., 2009). VR is often described as a set of technologies that enable people to immersively experience a world beyond reality (Berg and Vance, 2017). It has been used over the years within different fields of research, including education (Merchant et al., 2014), human resources (Alcañiz et al., 2018), medicine (Chicchi Giglioli et al., 2017; McGrath et al., 2018), neuroscience (Bohil et al., 2011), and psychology (Teo et al., 2016).

After reviewing 150 papers related to VR from 1994 to 2018 from the Web of Science (WOS), specifically VR in Marketing, Loureiro et al. (2019) concluded that two of the most studied VR sectors are tourism and retailing. Despite the vast literature covering the retailing field, however, there are several gaps concerning the use of newer technology such as VR and augmented reality (AR) in the retail environment (Moorhouse et al., 2018). VR has many barriers: for example, consumers are not yet familiar with wearing head-mounted displays (HMDs: McKone et al., 2016), and human interaction and communication are needed when integrating emergent technologies. Indeed, unfamiliarity with these technologies often leaves consumers apprehensive about trying them due to fear of incapability and usage complexity (Moorhouse et al., 2018). As a result of these issues, there is a slower adoption of newer technologies like Virtual Commerce (V-Commerce), in place of brick-and-mortar stores.

However, due to the current COVID-19 pandemic, the benefits of online shopping provisions are more prominent than they have ever been, for both consumers and retailers. While some businesses are struggling, some businesses are thriving. This is true for several Internet-based businesses, such as those related to online entertainment, food delivery, online shopping, online education, and solutions for remote work. A combination of government-imposed restrictions on movement and potential health and safety risks of using bricks-and-mortar shops, have made online trading an essential revenue stream for many retailers on a global scale (Schnack et al., 2021). This recent trend could also further accelerate consumer demand for shopping online (Donthu and Gustafsson, 2020), and is most likely to result in more focused business investments in online shopping platforms, including the development of immersive VR technologies for V-commerce.

Alcañiz et al. (2019) proposed that, in future, “two purchase channels will coexist: a channel with virtual stores, in which it will be possible to interact virtually with virtual sellers and products, and another channel with physical flagship stores, in which the consumer can have a real interaction with real sellers and products.” A recent prototype platform called Virtual Reality Online Shopping (VROS) was created to bring the physical shopping experience into the VR world by incorporating different online shopping stores (Huang and Liu, 2020). Virtual simulation environments add convenience and flexibility and increase the ability to scale and distribute simulations widely with lower costs. In conjunction, several other benefits like personalization of VR stores and recommendation systems (Pierañski and Strykowski, 2017; Walczak et al., 2019) are adding newer ways to help consumers in their purchase. This has led to the use of VR simulation environments for research in consumer behavior and marketing.

While immersive VR technologies provide retailers with alternative ways to market their products online, there remains a limited understanding of how consumers behave in such simulated environments and the factors that influence such behaviors. Consumer behavior research has identified internal and external factors as the key influences of consumers’ purchasing behavior. Internal factors include attitude, beliefs, demographics, feelings, lifestyle, motivation, and personality traits, whereas external factors include culture, locality, and the reference group (Sandhusen, 2000). Many previous studies have researched personality in consumer behavior, like brand preferences (Banerjee, 2016), impulsive buying behavior (Pelau et al., 2018; Sofi and Najar, 2018), online purchase intentions (Iqbal et al., 2021), and travel-related consumer-generated media (Yoo and Gretzel, 2011). Only a select few studies have focused on the personality of the consumer in a virtual environment, examining, for instance, shyness as a personality trait in a virtual world (Hammick and Lee, 2014), the effects of different interior architectural forms on emotional states by considering personality traits in a VR setup (Banaei et al., 2019), presence measure (Kober and Neuper, 2013), consumer attitudes in AR (Srivastava et al., 2021). A subset of those studies have focussed on Big Five personality like the effect of Big Five personality on optimal stimulation related to consumer innovativeness (Van Kerrebroeck et al., 2017) and the impact of Big Five personality traits on purchase behaviors (Schnack et al., 2021). Although the use of VR has recently increased, there remains a gap in research on the impact of the Big Five personality on consumer behavior in immersive VR, this study addresses this gap.

Research from the domain of automaticity proposes that the majority, if not all, of human behavior either begins as an unconscious process or occurs completely outside of conscious awareness (Martin and Morich, 2011). To date, most of the theoretical constructs used in consumer behavior and marketing are based on explicit measures, such as self-report questionnaires, interviews and projective measures (Alcañiz et al., 2019). Some effects, including data interpretation and subject knowledge (Chan, 2009) and social desirability (Grimm, 2010), have a negative impact on the reliability and validity of these techniques. The inclusion of physiological and neuroscientific approaches can advance consumer research by providing insights into the often unconscious mechanisms underlying consumer behavior (Bell et al., 2018). In recent years, several techniques have been proposed for the implicit measurement of consumer behavior, based on brain activity measures (Electroencephalography or EEG and functional Magnetic Resonance Imaging or fMRI), psychophysiological signals (e.g., eye-tracking or ET, skin conductance, and heart rate), and behavioral measures (e.g., navigation and product choice). This led to the creation of a new multidisciplinary field: consumer neuroscience (CN). CN has been poorly addressed using VR interfaces; most studies have used 2D non-immersive stimuli to examine CN (van Herpen et al., 2016; Ploydanai et al., 2017; Martínez-Navarro et al., 2019). The present study addresses this gap in the literature by using 3D immersive stimuli in a virtual environment using a consumer-grade HMD.

Human behavior tracking (HBT) is a captivating research field that offers a set of tools to measure human behavior. In this context, we use virtual environments (VEs) since it can be used with commercially available sensors to gather data about behavior of an individual. Studies (albeit few in number) have defined HBT as techniques that report features like the time a person spends on each task, consumer paths, seeking behavior, purchase behavior (Bigné et al., 2016), body movements (e.g., the head, the hands and the rest of the body) and product movements (Alcañiz et al., 2019). This study contributes to the literature by defining HBT as a standard behavioral measure to track the behavior of an individual through the natural walking-based movement in 2D (navigation), head and hand movements in 3D (posture), interaction with objects and the environment (interaction), and eye movement in 3D (ET). Although ET is generally considered a physiological measure, the gaze movement is a type of pupil behavior. ET has several advantages: it is portable, non-invasive, simple to use and relatively inexpensive (Alvino et al., 2020). Traditional ET systems typically consist of a camera and IR source positioned below the stimulus area, most often a computer screen. Development of VR glasses for immersive virtual experience led to a difficult scenario for tracking eyes using traditional ET systems. Since the recently developed HMDs have an integrated ET system (e.g., VIVE Pro), this allows researchers to study the eye gaze behavior inside an immersive VR environment. This study therefore proposed a synergy between ET and HBT; the two have been used in combination to study brand choice and purchase decisions in virtual stores (Bigné et al., 2016). All the behavioral measures are solely based on the signals received from the VR hardware: namely, HMDs and controllers, without the need of any external wearable sensors measuring, for example, electrodermal activity (EDA), EEG, or heart rate variability (HRV). This study provides a comprehensive comparison of these signals and their effect on a consumer’s behavior and specifically their personality.

The aim of this study was to highlight how the different Big Five personality traits are correlated with behavioral signals collected from VR gear, therefore helping researchers and marketers to understand personality of the consumers for future recommendation systems. This study thus attempts to bridge the literature gap by emphasizing the effect of the Big Five personality on consumers’ behavior in an immersive virtual hypermarket.

## Literature Review

### Personality

Over the past several decades, scholars have propounded multiple perspectives and iterations based on identifying and defining an individual’s personality. In the 1980s, a five-factor structure, known as the Big Five (Goldberg, 1981) or the five-factor model (FFM: McCrae and Costa, 1987), emerged and was considered more or less sufficient to encompass the trait-descriptive terms of personality. This five-factor structure putatively covers much of the covariation among self-ascriptions and peer ratings of personality descriptors (Benet and Waller, 1995). Although some of the specific factor labels have changed, the underlying composition has remained stable (John and Srivastava, 1999).

Most measures of individual differences in people’s behavior consider the same five factors or domains, in part or as a whole (Digman, 1990; McCrae and John, 1992; Tupes and Christal, 1992). The most common labels for these domains are extraversion, agreeableness, conscientiousness, negative emotionality (alternatively labeled neuroticism vs. emotional stability), and open-mindedness (alternatively labeled openness to experience, intellect, or imagination; Goldberg, 1993; John et al., 2008; McCrae and Costa, 2008). Extraversion indicates how outgoing and social a person is. Its traits include sociability, assertiveness and high energy levels. Agreeableness refers to how tactful, friendly, and warm a person is; its characteristics include compassion, respectfulness and trust. Conscientiousness relates to an individual’s level of self-discipline, and its traits include organization, productiveness and responsibility. Negative emotionality concerns a person’s ability to remain stable and balanced, and the associated characteristics include anxiety, depression and emotional volatility. Finally, open-mindedness indicates how open-minded a person is. Its traits include intellectual curiosity, aesthetic sensitivity and creative imagination.

Several questionnaires assessing the traits and features of each of the Big Five domains have been developed over the years. These include the NEO Five-Factor Inventory (NEO-FFI: Costa and McCrae, 1989), the Big Five Inventory (BFI: John et al., 1991), and BFI-2 (Soto and John, 2017b). Scholars have since created shorter, time-saving adaptations of these questionnaires, including a 10-item adaptation of BFI (Rammstedt and John, 2007), a 30-item BFI-2-S and a 15-item BFI-2-XS (Soto and John, 2017a). Personality has emerged as a highly influential factor in a wide range of contexts, including job performance (Tett and Burnett, 2003; Judge and Zapata, 2015) and job burnout (Swider and Zimmerman, 2010), mobile applications to assess personality traits (Xu et al., 2016), neuroscience and brain structure (DeYoung et al., 2010) and pro-environmental behavior (Kvasova, 2015). The relationship between a person’s personality and their consumer behavior has been a much-debated topic over the last century (Kassarjian, 1971; Foxall and Goldsmith, 1988). Despite having been researched extensively, however, the connection between a consumer’s personality and their behavior can still provide new insights into different topics; therefore, several authors have argued that the study should be revitalized (Bosnjak et al., 2007; Solomon, 2010).

### Consumer Behavior and Shopper Personality

Consumer behavior and marketing research consider shopper personality an essential factor to study its impacts on shopper attitudes and behaviors in particular marketplace settings (Roy et al., 2016). Shopper personality has been shown to influence shopping motives that impact the time a shopper is willing to spend in a store (Boedekar, 1995; Mehta et al., 2014) and a store environment’s perceived pleasantness when shopping (Kaltcheva and Weitz, 2006). Moreover, shopper personality has shown to define shopping needs (Mooradian and Olver, 1996) and consequently a shopper’s specific requirements in different retail settings.

Several studies have found a strong relationship between the Big Five personality domains and brand preferences (Banerjee, 2016), impulsive buying behavior (Pelau et al., 2018), online behavior (Dobre and Milovan-Ciuta, 2015), political consumer behavior (Quintelier, 2014), sustainable consumer behavior (Luchs and Mooradian, 2012) and travel-related consumer-generated media (Yoo and Gretzel, 2011). Moreover, while extraversion, conscientiousness and openness to new experiences have a positive influence, neuroticism negatively affects intentions to purchase from global brands (Zabkar et al., 2017). Dobre and Milovan-Ciuta (2015) highlighted that in an online shopping context, anxious people attach great importance to warranties and ensuring the confidentiality of operations, while extroverts focus on social opportunities, interactivity and store aesthetics. These personality traits are implicit in nature and do not easily change over time; thus, one can generally observe them through a consumer’s behavior.

Despite the previously discussed body of shopper research that suggests an impact of the Big Five personality traits on behavior of consumer, this relationship has not been extensively examined in an immersive VR context. To the best of our knowledge, only one study has researched this relationship (Schnack et al., 2021). Thus, it seems important to understand how consumer personality plays out in immersive VR shopping environments and how immersive VR environments can be designed to maximize shoppers’ dwell time, number of product purchases and more long-term outcomes such as store re-visits or word-of-mouth publicity.

### Behavioral Measures of Consumer Research

Consumer behavior can be traced using behavioral measures such as eye movement, store navigation, product interaction and body movement. ET has a rich history of research in a wide range of contexts, including, in particular, consumer behavior, due to its non-invasiveness. Several studies have used ET to examine, for example, the effect of smell on eye-catching behavior and memory (Tomono et al., 2011), information processing and attention (Pfeiffer et al., 2017), customer’s impulsivity (Moghaddasi et al., 2021), perceived realism (Carlson et al., 2011; Meißner et al., 2017) and perceived presence (Tonkin et al., 2011), product or brand choice (Siegrist et al., 2019), and purchase decisions (Bigné et al., 2016). Khatri et al. (2020) also used eye gazes to classify consumers’ ages in a virtual store.

Previous studies have suggested that approximately 80% of a shopper’s in-store time is spent navigating, and the remaining 20% is spent deciding which items to purchase (Sorensen, 2016), making navigation an important factor in deriving consumers’ purchase behaviors. Different types of navigation measures have been studied in real-store contexts. In-store behavior has been determined *via* grocery store shopping paths and purchase behavior (Hui et al., 2009), and other studies have focused on navigational web atmospherics (Dailey, 2004) and the effects of competition and cooperation in navigation behavior and spatial memory recall (Liang et al., 2019). Shen et al. (2015) used RFID technology to capture customers’ in-store behavioral data *via* indoor mapping and navigation of a real supermarket. Sorensen et al. (2017) argued that most of these shopping trips are short and shoppers only cover a small area during a given shopping trip; the authors also observed similar patterns in the number of items bought.

In addition to navigation, interaction techniques in the context of shopping activities have also been studied in a virtual environment (Verhulst et al., 2016). Interaction generally refers to the metaphor used to interact with the products in the VE but specifically interaction with products can be with defined as measures such as the number of items picked up, put down, and purchased. Along with other variables, the number of items purchased has been used to study in-store emotional states (pleasure and arousal) in a retail environment (Sherman et al., 1997). Posture has also been considered, and researchers have analyzed the role of pupil dilation in predicting preferences, judgments and choices (Ramsøy et al., 2017). While behavioral measures such as ET, navigation (NAV), posture (POS), and interaction (INT) have been thoroughly examined, very few studies have used these measures to assess consumer personality.

### Tasks and Interaction Analysis in Virtual Reality Shopping

Segmentation of data can facilitate the processing and analysis of data in CN. Since, some interactions happen in specific zones and regions as well as separate tasks, it is important to segment the data based on tasks and regions. Following subsections will describe the different tasks and regions of interest (ROIs) considered for segmentation of the data.

#### Free Exploration vs. Directed Navigation

In a consumer behavior scenario, most existing VR studies have allowed the consumer some degree of freedom while performing tasks. For example, consumers had the freedom to choose between a limited number of products within a budget (Ketelaar et al., 2018; Zhao et al., 2018); they could select a sequence of items without considering a budget (Verhulst et al., 2016; Liang et al., 2019), or they could choose preferred products (Meißner et al., 2017). Some investigations have allowed participants to undertake a series of search tasks (Liu and Uang, 2016) and brand choices (Martínez-Navarro et al., 2019), to navigate freely and look around the virtual environment (Van Kerrebroeck et al., 2017), and to browse the virtual products (Pantano and Laria, 2012; Wong Lau et al., 2014; Lau and Lee, 2019). Other studies, meanwhile, restricted the participants by putting them in a specific hypothetical situation, such as selecting cereal for a kids’ camp and a friend on a low-sugar diet (Siegrist et al., 2019). Speicher et al. (2018) administered an exploration task without an explicit goal before each search task, allowing participants to browse the environment, obtain information about the rooms, orient themselves to the world and build up knowledge. Some studies permitted the participant to roam freely before reaching the shelf and purchasing products such as beer (Bigné et al., 2016) or fruit and vegetables (Verhulst et al., 2017). There is much to be gathered from exploration tasks regarding consumer behavior because individuals behave differently or, rather, naturally, when exploring at their own pace. The present study adds to the existing literature by comparing the exploration task and two specific directed search tasks and analyzing their effect on the personality of the consumer.

#### 3D Regions of Interest

Since the recorded data is raw and unstructured, extracting structured features from these raw signals mostly follows two approaches. The first involves general features, which are not related to any specific zones but rather the whole shopping period. The second focuses on zonal features, which are related to a particular period in the task when the shopper is inside a zone on the floor plan (known as a zone of interest or ZOI) or is interacting with or looking at a specific area at the shelf level, known as an area of interest or AOI (Moghaddasi et al., 2020). Additionally, these characteristics can be sub-divided into features related to space, time and kinematics inside the VR environment. This study expresses the differentiation in these features to show the effect of a consumer’s personality on different structured features, including zonal vs. non-zonal and compares spatial, temporal and kinematic feature types.

### Research Questions

This study’s primary objective was to classify shoppers with different personality based on their behavior while performing different tasks in a virtual environment. Secondarily, to determine which behavioral measures can be used to improve classification of personality domains. A virtual store was created and several machine learning methods were applied to address the following research questions:

RQ1: To what extent can the personality of the consumer be recognized from their behavior while shopping in a VS?

RQ2: Do consumers with specific personalities show a similar pattern in their behaviors, and which scenarios can elicit these behaviors?

RQ3: How different type of features (spatial, temporal, or kinematic) impact or improve the discrimination of various domains of personality?

RQ4: Which combination of signals (ET, NAV, POS + INT, and HBT) and tasks (Free Exploration vs. Directed Navigation) can improve the recognition of personality of consumers in an immersive VS?

## Materials and Methods

### Participants

A convenience sample of 60 participants was recruited through an agency. Participants had to meet the following criteria: aged between 18 and 36 years old; no motor diseases; no evident mental pathologies; normal or corrected to normal vision and hearing. Three participants were excluded due to non-usable, corrupted ET data. All analyses were performed on the final sample, which consisted of 57 participants (47% women and 53% men; mean age = 25.19, *SD* = 5.06). Previous level of VR experience was recorded where 46% had no experience, 53% had experienced it once and 1% had experienced VR multiple times. All the participants received a financial incentive for partaking in the experiment, regardless of whether their data was used. The study was approved by the Ethical Committee of the Polytechnic University of Valencia with written informed consent from all participants in accordance with the Declaration of Helsinki.

### Personality Assessment

The personality assessment was conducted using the BFI-2-S that is a shortened version of BFI-2 (Soto and John, 2017a). This measure is composed of 30 items. Respondents rated each item using a 5-point Likert scale ranging from ‘disagree strongly’ to ‘agree strongly.’ At the level of the Big Five domains, the BFI-2-S retains about 90% of the BFI-2 domain scales’ reliability, self-peer agreement, and external validity (Soto and John, 2017a). Considering the time of the study spent in a virtual environment along with the sample size, BFI-2-S was selected as a domain level scale of personality for this study to reduce respondent fatigue.

In this study, the shoppers were divided into two categories based on the median of total scores. The total scores were calculated by averaging the scores of the answers. Note that the scores for 3 questions (out of 6) in each domain were reversed by subtracting the score from 6. To test the robustness of the validated BFI-2-S questionnaire with the current sample and translation, several tests were conducted. Cronbach’s alpha was used to assess the internal consistency of the five domains of BFI-2-S. A Cronbach’s alpha value between 0.7 and 0.95 is acceptable in most studies (Bland and Altman, 1997). A low alpha value could be due to a low number of questions, poor inter-relatedness between items or heterogeneous constructs. Bartlett’s test of sphericity was conducted to measure if the observed correlation matrix is an identity matrix. Significant *p*-value is desired to indicate that the correlation matrix is not an identity matrix (Bartlett, 1951). Kaiser–Meyer–Olkin (KMO) Test was conducted to measure the suitability of data for factor analysis. A KMO > 0.9 was marvelous, in the 0.80–0.70 s, middling, in the 0.60s, mediocre, in the 0.50s, miserable, and less than 0.5 would be unacceptable (Kaiser and Rice, 1974).

### The 3D VR Store Environment

The virtual environment was created with a Unity 3D game engine (Unity, 2020) and comprised a 6 m × 6 m virtual store (VS) and a training room of the same size. The virtual space corresponded to the physical space, which allowed for the free movement and natural walking style of the participants in the real world. The virtual simulation was run on Steam VR (SteamVR, 2020) by Valve Corporation, paired with HTC Vive Pro (2020) HMD, controllers, and four base stations, one in each corner of the room.

3D movement data, along with axis rotation data, was recorded from HMD for the head-tracking element. Similarly, hand tracking, or the 3D movement data of hands, was recorded using the controller. HTC VIVE Pro includes an inbuilt ET system which uses infrared sensors and emitters. It has a lens resolution of 1,440 × 1,600 pixels per lens (2,880 × 1,600 pixels combined), with a field of view of 110°. The raw gaze data was collected at a variable sampling rate of 60–70 Hz using HTC SRanipal SDK.

#### Virtual Store

**FIGURE 1:**
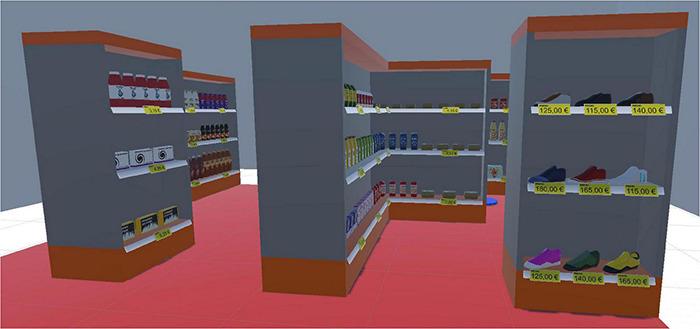
Virtual store with seven shelves and three shelf levels.

The VS consisted of seven shelves with three levels each (upper, middle, and bottom), as seen in Figure 1. Each shelf contained realistic product models of fast-moving consumer goods, such as milk, juice, coffee, and noodles, as well as durable goods (e.g., shoes). The products were highly interactable: they could be picked up, rotated, dropped, and purchased (if applicable). A blue circle in one of the corners was used as a trigger point to start and end tasks.

#### Training Room

**FIGURE 2:**
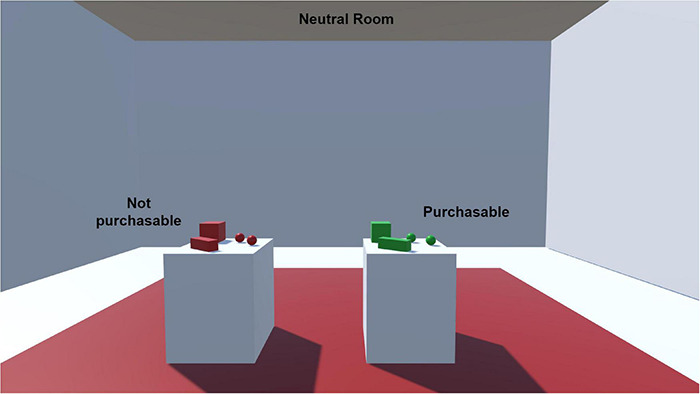
Training room showing purchasable and non-purchasable objects.

The training room contained two white tables at its center. Each table held four objects of different shapes (sphere, cube, and rectangle): green-colored items were on one table, and red-colored items were on the second, as shown in Figure 2. The objective of the familiarization task was to allow the participant to become familiar with the technology by learning to move in the environment and use the controllers. Participants were asked, for example, to pick up green objects, rotate them to see all sides and hold the purchase key for 3 s to buy the item; after a successful purchase, the object vanished, accompanied by a soft sound. Similarly, users were asked to pick up red objects, rotate and try to purchase them. Since the red items were not purchasable, however, a buzzer sound informed them as such. Simple items of green and red color were used to avoid bias effect of experimental products. Participants were also asked to take a walk around the room to be familiar with navigating in a virtual environment while wearing HMD.

### Protocol

The experiment was conducted at the LENI laboratory of the Polytechnic University of Valencia. All participants performed all tasks (within-subject design) in the same sequence. Upon arrival at the laboratory, participants were welcomed and seated, and the research procedure was explained to them. After reading and signing the informed consent form, participants were taken to the starting point of experiment to wear the HMD with the experimenter’s help. All participants started at the same point (a blue circle in VS) facing the same general direction. The familiarization task described above was conducted in the training room, where participants were informed about the mechanics of the VR gear and the controllers. When the 4-min time limit was over, or when participants became familiar and comfortable with the VR gear’s mechanics, participants were instructed to go to the blue circle at the corner to finish the task. After the familiarization task, participants underwent a calibration for the eye tracker following HTC routines. Following the calibration, participants were presented with instructions on the screen. If they were not able to read the instructions, the headset was removed, lenses cleaned and set up on the participant again with ET calibration. After receiving instructions, participants started the tasks, each of which is detailed below.

#### Task 1 (Exploration Task)

Participants were instructed to roam freely and explore the virtual store for up to 4 min in this task. They could interact with the products present in the store and could end the task early by standing on the blue circle.

#### Task 2 (Search for and Buy Snacks Task)

In this task, participants were asked to search for the shelf containing snacks (potato chips) and purchase the ones of their choice. The shelf held nine snacks in total (three snacks of different types and prices on each level). There was a limited budget of 5 Euros given to each participant; since the snacks were priced between 1.25 and 3 Euros, they had the choice to buy up to three snacks because the price added up to 4.25 Euros for the cheapest three snacks. After making their purchases, they were instructed to return to the blue circle to finish the task.

#### Task 3 (Search for and Buy Shoes Task)

Much like Task 2, participants were instructed to search for the shelf containing shoes. There were nine shoes of different colors and prices distributed over three shelf levels. The shoes ranged in price from 115 to 180 Euros; since participants had a budget of 180 Euros, they could only choose one pair of shoes. After buying the shoes, they were instructed to return to the blue circle to finish the task.

The difference between the search for and buy tasks is the type and the price of the products. The products in the first forced search task considered daily use products with cheaper price, but in the second forced search task, the product was hedonic with higher price. Following Task 3, participants removed the HMD and completed questionnaires, including the BFI-2-S questionnaire.

### Data Segmentation

**FIGURE 3:**
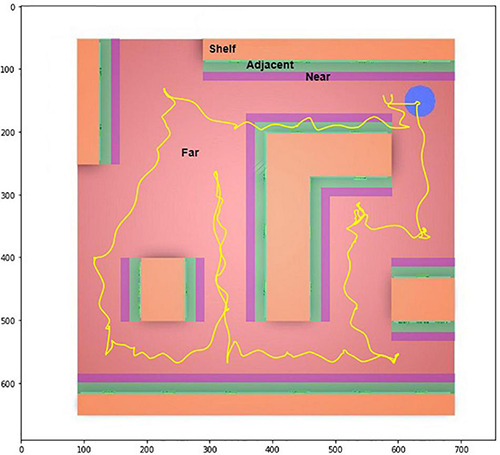
Zenithal view of the virtual hypermarket showing ZOIs and navigation of a participant.

**FIGURE 4:**
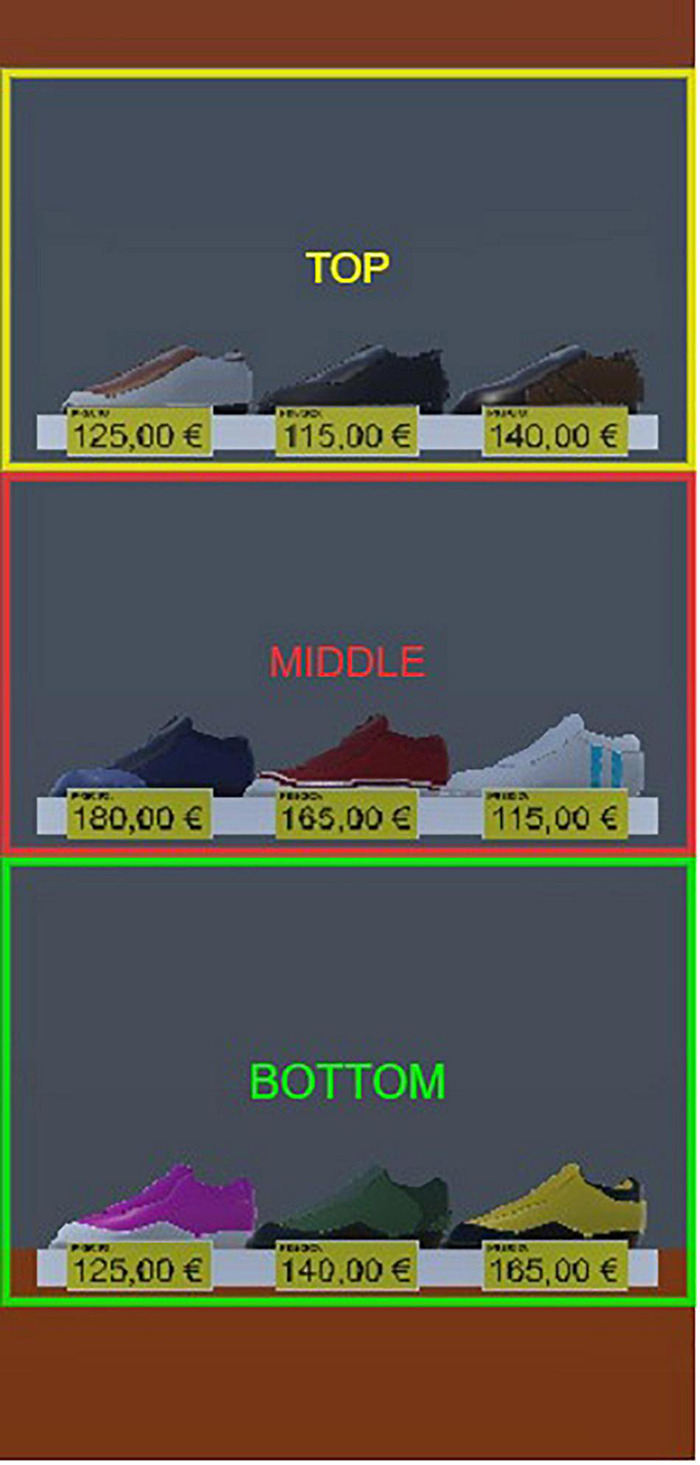
Shelf with three levels of AOIs for Tasks 2 and 3.

From a zenithal perspective, the 2D VS space was segmented into four zones (ZOIs). These 2D-ZOIs were defined based on their distance from the shelves, dividing the floor plan into different zones. As shown in Figure 3, ‘shelf’ is orange, ‘adjacent’ (next to shelf) is green, ‘near’ (next to adjacent) is purple, and ‘far’ (remaining explorable areas) is in red. The figure also shows the movement of a participant during the exploration task, taking the blue circle as the starting and ending point. Similarly, for the shelf, adjacent and near ZOIs, the 3D space (including height) was segmented into three AOIs. As seen from a first-person perspective, the top level of the shelf was in ‘shelf-top’ AOI; the middle level of the shelf was in ‘shelf-middle’ AOI, and the bottom level was in ‘shelf-bottom’ AOI, as shown in Figure 4. Similarly, these levels were applied to the adjacent and near ZOIs, but the ‘far’ ZOI was excluded from the 3D segmentation.

**FIGURE 5:**
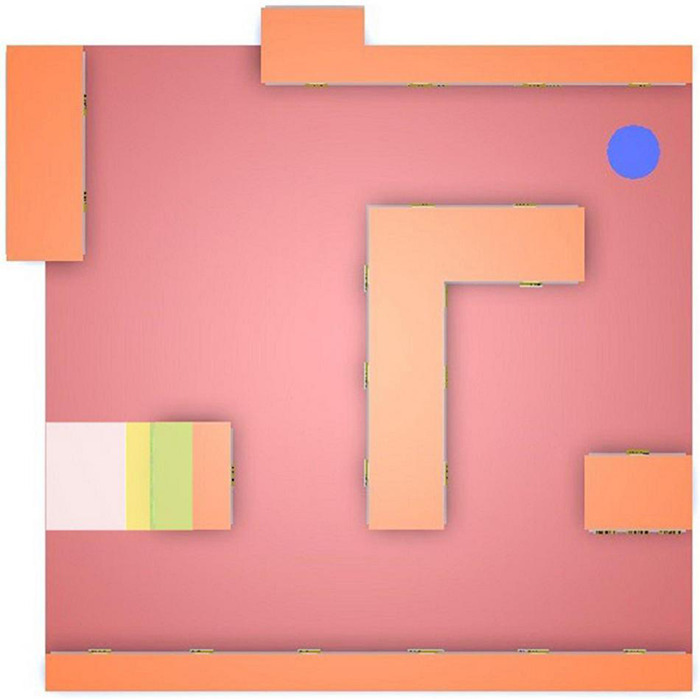
Zenithal view of zones and areas of interest for Task 2.

**FIGURE 6:**
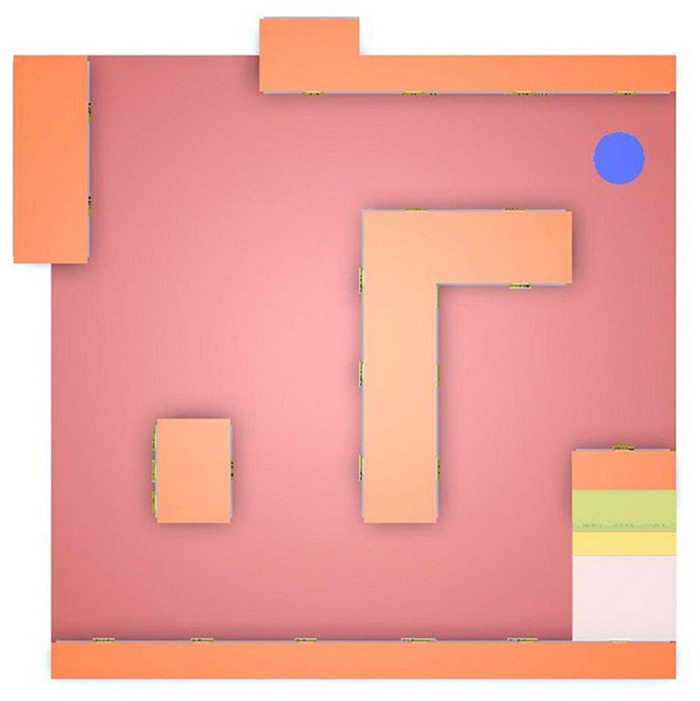
Zenithal view of zones and areas of interest for Task 3.

In the case of Tasks 2 and 3, since the shelf of importance was the one pertaining to the task (i.e., the target shelf), the ZOIs were divided to reflect this, as shown in Figure 5 for Task 2 and Figure 6 for Task 3. In these tasks, the shelf and the area in front of the shelf were classified as shelf, adjacent, and near ZOIs, and the rest of the store area was considered far ZOI.

### Data Recording and Pre-processing

Data pre-processing and analysis were done using Python version 3.7.3 in the Jupyter environment. The scripts were written using Numpy, Pandas, Matplotlib, scikit-learn (Pedregosa et al., 2011), and Pickle libraries.

The data were recorded using HTC VIVE’s input devices (namely HMD and controllers). Raw data from the simulation was converted into four modules using various pre-processing techniques, including the segmentation of ZOIs.

1. ET: ET data pre-processing from gaze data for fixation and saccade classification was conducted using the dispersion-threshold identification (I-DT; Salvucci and Goldberg, 2000) algorithm in a 3D environment. The parameters were set as follows: the mean time fixation was at 0.25 s, and dispersion threshold less than 1° (Llanes-Jurado et al., 2020). Every duration and centroid were computed for each fixation.
2. NAV: NAV data were pre-processed using the raw movement data from head tracking. It considered the movement of the participant in two dimensions (*x* and *z*) and did not consider the height (*y*) dimension (i.e., it considered ZOIs and not AOIs).
3. POS: Similarly, POS data was pre-processed using raw movement data from head and hand tracking but considered the participant’s movement in all three dimensions. It considered both AOIs and ZOIs.
4. INT: INT data consisted of the simulation events (e.g., start and end times, time at which an object was picked up, and the number of items picked up).

Due to a smaller number of features related to interactions, we combined posture and interaction as POS + INT. A combination of all these pre-processed data (ET + NAV + POS + INT) made up the HBT module. In addition to the HBT module, the BFI-2-S questionnaire data were analyzed using the scoring method to produce a domain-specific score for each participant.

### Data Analysis Using Machine Learning

**FIGURE 7:**
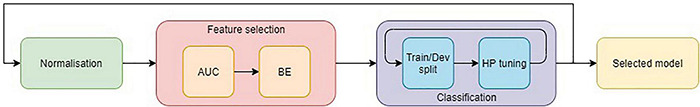
Model selection pipeline for machine learning.

A supervised machine learning (ML) pipeline was created for analyzing data using scikit-learn python library. Training in supervised ML algorithm is performed using some observation samples with ground truth labels. In this study, the labels corresponding to the classes are the high and low levels of each domain. To differentiate these two levels, the median method which creates balanced classes and distinctive population centers was used. It was necessary to extract distinctive features such as fixation duration, velocity of movement, number of products purchased, etc. (kindly look at the Supplementary Appendix A for the full list of features). Here, the features are extracted by some physical definitions considering the type of data (Figure 7). After feature extraction, the features were used for classification after a simple pre-processing, normalization and feature selection phase.

#### Pre-processing and Normalization

At first, the features that did not contain any useful data (such as features with all zeros or the same elements) were removed. Furthermore, the features that were linearly dependent on each other were removed using Pearson correlation coefficient with 0.95 as the threshold. Due to this, the features which did not contain new information were removed. Then a min–max normalization or rescaling was performed to map the features between zero and one.

#### Feature Selection

Since the number of features was substantial and some of the features might not have been as informative as others, some features using area under the curve (AUC) filtering method were removed. The number of features after this step was set to become 50. AUC was applied to signals with more than 50 features; the NAV data had less than 50 features after normalization and thus skipped the AUC step. After AUC, a backward elimination (BE) algorithm was applied to remove more features from this set, making the count of selected features 10 or below. BE ranked the features using an SVM classifier with *K*-folds cross-validation method with 10-fold (Efron, 1983). These steps were necessary to avoid overfitting the classifier to the training set.

#### Classification

The SVM method was used for the binary classification problem in the current work (Chang and Lin, 2011). Here, a cross-validation method, i.e., stratified *K*-folds cross-validation with 10-fold, was used. In this method, each participant has the opportunity to be used for testing the model trained with the rest of the observations. The folds helped to reduce the impact of diversity in the distributions of the testing and training data and tune the hyper-parameters. In an SVM model, some hyperparameters, such as the type of kernel, regularization parameter, gamma in the ‘RBF’ kernel, degree, and gamma in the polynomial kernel, should be tuned. This tuning/optimization is the process of searching for the best parameters of a model so that the model can optimally solve the ML problem. Besides, to reject the effects of variability, this procedure was repeated in 50 different runs. In the end, the average of the accuracy for all the repetitions was reported as the prediction accuracy.

## Results

### Self-Assessment Observations

Applying the method outlined in the personality recognition section, the observations were divided into two significantly different groups based on their *p*-values in Table 1. Based on Table 1, the groups were balanced, and the centers for the populations in each group were different. The Cronbach’s alpha for the agreeableness domain was 0.546: close to 0.5 which is fairly low. The other domains were within the range of 0.65 (barely acceptable) and 0.8 (good consistency), with negative emotionality showing the best internal consistency as shown in Table 1. Bartlett’s test of sphericity shows significance through *p*-value (<0.001). The test was statistically significant, indicating that the observed correlation matrix is not an identity matrix. KMO value when considering all domains of personality was 0.49, when considering 4 domains excluding agreeableness, it reached 0.59. The results of factor analysis can be seen in Supplementary Appendix B where a 5-factor solution explained a total of 44% of the cumulative variance (Supplementary Appendix Table B3). In Supplementary Appendix Table B1, factor analysis shows a good correlation with all questions with respect to their domains. Each domain had 3 direct and 3 reverse questions which can be seen with negative correlation within each domain. Supplementary Appendix Table B2 shows the aggregated results of question sets for all domains; factor loading shows Negative emotionality with the best loading with aggregate 0.60 followed by Extraversion, Conscientiousness and Open mindedness with each approximating around 0.52 aggregate. It can be seen that agreeableness has significantly low factor loading in aggregated results (0.25). Thus, factor analysis and Cronbach’s alpha indicate that 4 out of 5 factors are robust where agreeableness results were extrapolated carefully for the current sample.

**TABLE 1:** Labeling results and Cronbach’s alpha of all BFI-2-S domains.

Domain	Balance	Group centers (high: low)	*P*-value	Cronbach’s alpha
Extraversion	27: 30	4.03: 3.05	<10^–6^	0.684
Agreeableness	27: 30	4.36: 3.47	<10^–6^	0.546
Conscientiousness	28: 29	4.04: 2.97	<10^–6^	0.691
Negative emotionality	24: 33	3.61: 2.26	<10^–6^	0.800
Open-mindedness	27: 30	4.32: 3.29	<10^–6^	0.723

### Personality Recognition

The accuracy of the ML model was calculated by comparing the predicted labels with the ground truth labels from the results of the BFI-2-S questionnaire for supervised learning. Different tasks attained different accuracy levels for all signals, as shown in Table 2. In Tasks 1, 2, and 3 independently, extraversion and conscientiousness showed better accuracies in exploration with an accuracy of 0.74 and 0.71, respectively, for the combination of signals (HBT). Meanwhile, negative emotionality presented a better accuracy in the directed search tasks, achieving an accuracy rate of 0.73. However, agreeableness and open-mindedness could be recognized in both exploration and directed search tasks, reaching more than 0.70 accuracy in both task types.

**TABLE 2:** Accuracy of classification of personality domains over all iterations in tasks and signals.

Task(s)	Signal	Extraversion	Conscientiousness	Agreeableness	Negative emotionality	Open-mindedness
1	ET	0.65 (0.05)	0.63 (0.04)	**0.77 (0.04)**	0.60 (0.04)	0.65 (0.05)
	NAV	0.63 (0.03)	0.66 (0.03)	0.65 (0.03)	0.55 (0.04)	0.55 (0.01)
	POS + INT	**0.72 (0.04)**	0.66 (0.03)	**0.72 (0.03)**	0.62 (0.04)	0.67 (0.05)
	HBT	**0.74 (0.03)**	**0.71 (0.03)**	**0.78 (0.03)**	0.67 (0.03)	**0.75 (0.05)**
2	ET	0.58 (0.01)	0.62 (0.04)	0.62 (0.01)	0.64 (0.04)	**0.72 (0.03)**
	NAV	0.58 (0.00)	0.56 (0.02)	0.62 (0.01)	0.64 (0.03)	0.65 (0.03)
	POS + INT	0.69 (0.04)	0.62 (0.05)	**0.75 (0.03)**	0.66 (0.04)	0.69 (0.04)
	HBT	0.69 (0.04)	0.67 (0.05)	**0.76 (0.03)**	**0.72 (0.04)**	**0.76 (0.03)**
3	ET	0.61 (0.03)	0.60 (0.04)	**0.72 (0.03)**	0.67 (0.03)	**0.73 (0.04)**
	NAV	0.58 (0.02)	0.58 (0.02)	0.62 (0.02)	0.60 (0.04)	0.55 (0.03)
	POS + INT	0.61 (0.04)	0.68 (0.04)	**0.72 (0.03)**	0.67 (0.03)	0.67 (0.04)
	HBT	0.61 (0.05)	0.64 (0.04)	**0.76 (0.03)**	**0.73 (0.03)**	**0.77 (0.03)**
1, 2, 3	ET	**0.70 (0.05)**	**0.74 (0.04)**	**0.81 (0.03)**	**0.76 (0.03)**	**0.81 (0.03)**
	NAV	0.64 (0.03)	**0.71 (0.04)**	0.66 (0.03)	0.67 (0.04)	0.67 (0.04)
	POS + INT	**0.72 (0.04)**	**0.76 (0.04)**	**0.80 (0.03)**	**0.74 (0.03)**	**0.75 (0.03)**
	HBT	**0.79 (0.04)**	**0.81 (0.04)**	**0.85 (0.03)**	**0.80 (0.03)**	**0.79 (0.03)**

*Models with more than 0.70 accuracy are highlighted in bold.*

Regarding separate signals, ET was more informative measure for the prediction of agreeableness and open-mindedness domains, achieving an accuracy of 0.77 for agreeableness in task 1 and 0.73 for open mindedness in task 3. Similarly, POS + INT showed a greater discrimination capability for the prediction of extraversion and agreeableness domains achieving an accuracy of 0.72 for both. Conversely, NAV was the weakest measure among all the signals to predict the domains in all tasks, while conscientiousness and negative emotionality showed coherence only with combination of all signals (i.e., with HBT).

With putting the extracted features from all the three tasks together, the accuracies improved for all domains in all the signals except NAV. All the domains are best recognized considering the accuracy *via* a combination of signals (HBT), achieving an accuracy level of 0.78 for extraversion, 0.81 for conscientiousness, 0.85 for agreeableness, 0.80 for negative emotionality, and 0.79 for open-mindedness.

### Selected Features

**FIGURE 8:**
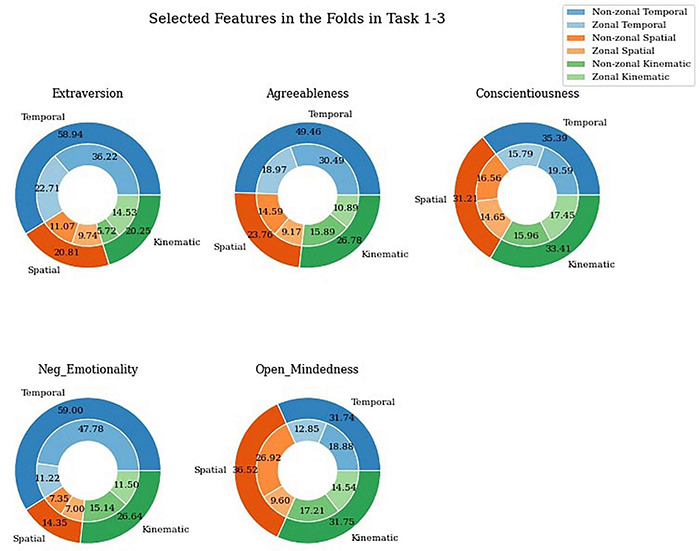
Selected temporal, spatial, and kinematic features between tasks.

With respect to temporal, spatial and kinematic feature types, Figure 8 shows the relationship between all personality domains in all tasks with respect to selection of HBT features. Extraversion, agreeableness and negative emotionality showed a stronger relationship with temporal type with 58.94, 49.46, and 59% selected features, respectively. Spatial features on the other hand, were selected more in conscientiousness and open mindedness at 31.21 and 36.52%, respectively, almost similar to kinematic features at 33.41 and 31.75%, respectively, when compared to other domains. The difference in selection of spatial and kinematic features is <5% in all domains except negative emotionality where spatial is 14.35% and kinematic is 26.64%. Conscientiousness showed similar features for all three feature types [i.e., one-third for each with a slightly higher rate (∼4%) in the temporal feature type]. Open-mindedness reflected a comparable distribution among the three feature types, with a somewhat stronger relationship with the spatial feature type (∼4%).

When considering zonal and non-zonal features, non-zonal features were selected more in temporal features type in all domains of personality when compared to zonal features as 61.45, 61.64, 55.35, 80.09, and 59.48%, respectively, for extraversion, agreeableness, conscientiousness, negative emotionality, and open mindedness. Spatial features on the other hand have close to equal or more features selected for non-zonal features in all domains (53.19, 61.40, 53.05, 51.21, and 73.71%, respectively). Zonal features were selected more in kinematic section of extraversion and conscientiousness as 71.76 and 52.23%, respectively.

## Discussion

The primary objective of this study was to classify consumers based on the Big Five personality domains using their behavior while performing different tasks in a virtual shop. The secondary objective of the study was to highlight how the different personality traits are correlated with behavioral signals in an immersive virtual scenario. To answer the research questions, we investigated the impact of behavioral features on each domain separately considering tasks and feature types.

Individuals with high level of extraversion dimension are sociable and active, exhibit dominant behavior and seek sensations (Robu, 2007). In the exploration task, enough freedom was given to the participants to allow them to move at their own pace and interact with the products, thereby inciting excitement and newness. This resulted in an increase of visible extraversion behavior, as suggested by the higher prediction accuracy of extraversion in the exploration task. With every individual taking their own time in the exploration task, the temporal features were selected more for extraversion.

The agreeableness dimension is characteristic of altruistic, kind, modest (Saleem et al., 2011), sensitive and self-confident people (Robu, 2007). Personality domain such as agreeableness can be predicted with more than 90% accuracy using ET data while showing affective image and video stimuli (Berkovsky et al., 2019). In consistency with this research, our results show high accuracy with ET data in VR for classification of agreeableness domain. Automatic assessment of agreeableness has been correlated with different posture in human-robot interaction using supervised learning (Zafar et al., 2019). Our study shows that regardless of the task, agreeableness shows good accuracy for POS + INT features and shows improvement when tasks are taken together. On the other hand, possibly because of the absence of other people in the environment and lack of any social factor, NAV features were not descriptive of agreeableness for both tasks. Agreeableness has also been shown to impact a shopper’s fundamental shopping motivation (hedonic vs. utilitarian), specifically increasing the importance of utilitarian motivations as driver for purchases during online shopping (Tsao and Chang, 2010). Research has also found that utilitarian shopping values have a positive impact on information search, which implies that agreeable shoppers would spend more time acquiring additional product information (Schnack et al., 2021). This is in conjunction with agreeableness showing high selected features related to time.

Much like extraversion, people with the conscientiousness personality type were also recognized in the exploration task. This could be because, in the exploration task, these individuals preferred to follow a plan rather than act spontaneously; thus, their self-discipline and organizational behavior emerged to regulate and direct their impulses. This behavior is hard to track using eye gaze patterns, navigation and posture separately. However, when all the signals were combined in HBT, the difference between high and low conscientiousness became noticeable. To recognize conscientiousness personality domain, different types of features were selected as frequent as each other.

Neuroticism or negative emotionality is defined as a disruption in emotional stability through negatively charged emotional states (Robu, 2007). The more neurotic a person is, the harder it is for them to control their emotions and impulsive purchases (Tsao and Chang, 2010). Higher classification accuracy for negative emotionality was seen in the directed search tasks due to these tasks being instructed, and they were not allowed to do any impulsive purchases. Therefore, it was harder for the neurotic consumers to control their emotions, and subconsciously they behaved differently than less neurotic consumers. Although combination of all the signals (HBT) improved the accuracy for ET and POS + INT signals which could be considered as informative signals by combining all the tasks. It can be related to the features’ ability to combinedly bring out small factors related to emotions. Otherwise, separate signals could not attain acceptable results. According to our results, negative emotionality, like agreeableness, had more temporal chosen features. Since neuroticism has been shown to have a direct effect on utilitarian shopping values (Cardoso and Pinto, 2010), and utilitarian shopping values have a direct effect on product information (Tsao and Chang, 2010). It can be implied that negative emotionality effects gathering of additional product information and hence effect on temporal features.

The openness to experience domain characterizes individuals who are willing to consider different points of view and opinions (Dobre and Milovan-Ciuta, 2015). Previously, it has been shown that open mindedness was positively related with the processing of irrelevant information and negatively associated with the processing of central information (Agnoli et al., 2015). Considering this information, we can expect that one of the best signals to recognize open mindedness is ET. This has been confirmed in our results with the highest accuracy related to ET among all the three types of signals. In exploration tasks, we did not have focus information, all the information was peripheral. However, in directed tasks, there was some information to focus and purchase some product of interest. Therefore, it can be inferred that both peripheral and focussed information existed in the directed tasks. Hence, the ET signal could discriminate the open mindedness in the directed task but not in the exploration task. Besides, the combination of ET signal with the rest of signals could improve the accuracy but this improvement is not considerable for the directed tasks (around 5%), however, in the exploration task, this improvement is more significant (15%). For open mindedness, all three feature types had almost equal distribution in selected features, with spatial showing a slightly higher number due to spatial features being more related to adventurousness and creativity in exploring the environment.

### Theoretical Implications

There is an extensive body of research on consumer behavior in e-commerce industry (Hwang and Jeong, 2016), but little is known about the behavior of consumers in immersive VR retail store environment. Prior to this study, concept of behavior tracking using ET, navigation, posture and interaction inside VR simulations had not been addressed in the retail literature. However, Schnack et al. (2021) discussed potential benefits of objective measures of shopper behavior using immersive VR retail simulations, this study therefore addresses an existing gap in the retail literature about the role of consumer personality in immersive VR store environments by providing a ML model with good classification accuracies (>70%) for multiple signals and tasks, as shown in Table 2. This suggests that the personality of a consumer can be recognized from their behavior while shopping in a virtual store with good accuracy, hence answering RQ1. Besides, each domain of personality can be recognized with a certain task and signal, as discussed in previous paragraphs which gives an answer to RQ2. The benefits and challenges of using VR systems over physical retail stores have been extensively studied (Bressoud, 2013; van Herpen et al., 2016; Burke, 2018). Additionally, these VR systems promise high ecological validity (Meißner et al., 2017) and can also complement shopper behavioral data with a range of biometric measures such as EEG, ET, skin conductance or heart rate. However, these VR retail stores are subject specific and address only certain aspects of retail experience which is difficult to generalize. Since it is interesting to study different aspects of VR retail experience, this study provides the combination of different types of tasks and signals and tests the accuracy of classifying personality domains among them. Results suggest that the combination of all tasks and signals provides the best accuracy of classification for almost all the domains in turn answering the RQ4. Though factor analysis along with Cronbach’s alpha shows a good correlation with all questions with respect to their domains with a good cumulative variance, Agreeableness results were less robust. Similarly, along with the types of tasks, it is also interesting to study the different kinds of features that can be extracted from the behavioral aspect of VR experience. This study while putting light on RQ3, shows that the best type of feature to discriminate various domains of personality was temporal, followed by kinematic as seen in previous paragraphs for all domains except open mindedness. For open mindedness, this order is different meaning that spatial is more informative than the other types. Although, the difference between all the types in this domain is negligible. However, due to the nature of the “Big Five” literature in consumer research, almost all of the studies measured consumers’ behavioral responses *via* self-reported survey methodology which are heavily biased on their intentions to report. On the other hand, current study differentiates with the literature by presenting the consumers with virtual stimuli. The information about received from different tasks and features provides objective measures of consumer behavior while improving the ecological validity of behavioral responses in VR and thus reduce the dependency of behavioral data on self-report measures.

### Practical Implications

With the growing popularity of Metaverse, fueled by the Facebook’ name change to Meta in 2021, it is expected that the future of social media is on the track to be projected in virtual environments. Evidently, when a new technology becomes popular, it leads to the influx of companies investing and working to manage to produce and sell their products in the said new technology, for example physical retail to e-commerce (Burt and Sparks, 2003). The field of marketing is undergoing a major transformation from physical retail to e-commerce and the next step is V-commerce (Xi and Hamari, 2019). Many retailers have already started incorporating VR in their marketing activities like IKEA (e.g., a virtual visit to a kitchen before buying it), Coca-Cola (e.g., a virtual visit to a company’s factory) and Tesco (e.g., virtual visits to different store layouts). Similarly, eBay’s Shopticals and Alibaba’s Buy+ have significant potential to change existing marketing practices. The findings from this study informs retailers and marketers who consider use of immersive VS for V-commerce applications about the role of consumer personality and how it impacts behavior. The study highlights the type of tasks and features that can be used to classify consumers with character traits of different personality domains. Besides implications for marketers, the study showcases the potential of immersive VR scenarios for in-store consumer research. Physical store operators can use virtual simulations to test for different new store layouts, product assortments, and store atmospherics. These simulations provide a detailed analysis of behavioral observations such as attention to products and other environmental factors, purchase intentions and impulsive purchases at comparatively low cost and time by providing data related to ET, navigational patterns, posture and interactions with products. Additionally, academics interested in how human behavior is impacted in different scenarios can utilize these VR to produce different scenarios.

## Conclusion

Many recent studies in consumer behavior research have focused on physiological signals, such as EEG (Pandey et al., 2020), fMRI (Lee et al., 2020), and functional Near Infrared Spectroscopy (fNIRS) (Çakir et al., 2018) to study consumers’ behavior. Similarly, VR is one of the most promising retailing innovations and will revolutionize the consumer shopping experience in the future (Grewal et al., 2017). This study builds a base for studying consumer behavior in VR lab environments in a time- and cost-efficient way while still providing a high degree of control over many variables, which would not be feasible in a real retail store environment. In this study, we found that the behavior of the consumers can be an indicative of the personality traits of the consumers in a VS. Also, we found that several different signals such as ET, NAV, POS + INT, and HBT can be used to classify consumers with different personality characteristics. Furthermore, we showed that each signal has different capability to distinguish the level of each personality domain. The classification accuracy based on each domain was shown to be highest when combining Tasks 1, 2, and 3. Similarly, a combination of signals (HBT) produced a more accurate classification for all domains. This means that HBT is a better indicator of behavior in all domains than other signals examined separately. This study aims to define a base for HBT, which can be replicated in future experiments and thus encourages further utilization of VR environments for expanding current literature of consumer behavior research.

### Limitations

This study is one of the first to consider personality in VR and to suggest the exclusive use of VR signals for consumer behavior research. Our results show significant relationships between the type of task and the signals used with each of the Big Five personality traits. However, the features assessed were dependent on the retail store used, and future research in VR should validate these results in other spaces. The ground truth labels used in this study are from questionnaire BFI-2-S; nevertheless, as a self-report measure, its reliability is limited. In addition to this, the BFI-2-S is a domain-specific shorter version of a questionnaire, and its reliability is lower than that of the BFI-2. Additionally, factor analysis showed agreeableness domain to be less robust with respect to other domains, this could be due to limited participants and/or limitation of self-assessment. VS was intentionally kept limited and simple because of computational limit of the system implementing the simulation on an increased play space of 6m x 6m which required higher GPU consumption. The difference in the complexity of environment can limit the ability to extrapolate, even when this is not the main objective of the study. Lack of cross-balance between the participants and tasks might lead to a bias effect or learning effect. Considering two similar tasks (here, search tasks) could also lead to learning effect and should use cross-balance to minimalize this effect in future studies. The translation of the validated BFI-2-S questionnaire showed less robustness for agreeableness, this could be due to sample size, thus a bigger sample size is recommended. Due to technological limitations, the design of current VS might have restricted participants to follow a circular pattern in navigation in the store. In the future, it would be beneficial to implement the VS in a bigger play space to increase the number of shelves and multiple entrance/exit points to increase the choices of navigation patterns.

### Future Research

The VR shopping simulations used currently do not take the unique personality of an individual into account separately, which is a core factor that substantially affects a customer’s shopping journey. These simulations are predetermined, predesigned, provide very specific scenarios and do not consider the subject’s nature, character or behavior. In particular, overt characteristics such as age, sex, ethnic and cultural differences, or more complex factors, such as personality, can significantly affect the consumer experience. Future V-commerce applications must contemplate creating adaptive scenarios which can be adjusted during the simulation, in real-time, to each participant’s unique classification based on behavioral measures. Thus, the secondary objective of the study was realizing which features are effective in classifying these behavioral aspects which will help in determining the essential features required for creating real time applications. VR shopping may advance to the point where customers can shop from inside their rooms, without needing to go out. In this context, the only information received by marketers will be from VR hardware. This study focused on the information generated through VR hardware only, using only headset and controller data and thereby facilitating a research model for personalization and advertisements for marketers. Classifying consumers’ personalities will help marketers produce better and more personalized VR products. Several studies have used sensors such as ET (Bigné et al., 2016; Khatri et al., 2020), NAV (Liang et al., 2019), POS and INT (Ramsøy et al., 2017) to conduct an experiment in a VR environment. There might be an increase in VR shopping in the coming years due to technological advancements and reduced costs of consumer-grade VR headsets: this will result in marketers focusing on personalizing VR products for the consumer based on their behavior. This methodology can also help in building recommendation systems, personalized shopping and product placements, which can, in turn, be adapted according to the consumer’s personality. This will help marketers improve the purchase rate of products and help consumers ascertain the product of their choice according to their behavior. Future research could focus on general comparative behavior patterns, such as hedonic vs. utilitarian, or the effect of social cues vs. environmental cues, using behavioral signals received from a VR setup. Another interesting facet of future research could be to investigate specific domains of behavior, such as risk perception, enjoyment, focus, or confusion. The adaptation of previous studies conducted in a real environment to a VR environment using this methodology is also an exciting avenue for further research.

## Data Availability Statement

The raw data supporting the conclusions of this article will be made available by the authors, without undue reservation, to any qualified researcher.

## Ethics Statement

The studies involving human participants were reviewed and approved by the Ethical Committee of the Polytechnic University of Valencia in accordance with the Declaration of Helsinki. The patients/participants provided their written informed consent to participate in this study.

## Author Contributions

JK, JM-M, MM, JG, and MA devised the methodology and defined the experimental setup. JK and MM acquired, processed, and analyzed the experimental data. JK wrote the initial draft. All authors revised the manuscript and approved the final text.

## Conflict of Interest

The authors declare that the research was conducted in the absence of any commercial or financial relationships that could be construed as a potential conflict of interest.

## Publisher’s Note

All claims expressed in this article are solely those of the authors and do not necessarily represent those of their affiliated organizations, or those of the publisher, the editors and the reviewers. Any product that may be evaluated in this article, or claim that may be made by its manufacturer, is not guaranteed or endorsed by the publisher.
