# Supplementary material for: Recognizing Personality Traits Using Consumer Behavior Patterns in a Virtual Retail Store
Source: Front Psychol. 2022 Mar 11;13:752073. doi: 10.3389/fpsyg.2022.752073 (PMC8962833; doi:10.3389/fpsyg.2022.752073)
Supplement: Supplementary file 1 [file Data_Sheet_1.docx]

Supplementary Material

# Appendix A

Table A 1 Full list of features extracted from raw data

| **Signal** | **Type** | | **Feature** | **Signal** | **Type** | | **Feature** |
| --- | --- | --- | --- | --- | --- | --- | --- |
| Eye-tracking | General | Temporal | Average Time of Fixations | Posture & Interaction | General | Temporal | Hand Average of Stops Duration |
|  |  |  | Average Time of Saccades |  |  |  | Hand Total Time |
|  |  |  | Percentage of Time for Fixations |  |  |  | Head Average of Stops Duration |
|  |  |  | Long Fixations |  |  |  | Head Total Time |
|  |  |  | Short Fixations |  |  | Spatial | Hand Total Distance Travelled |
|  |  |  | Total Time |  |  |  | Head Total Distance Travelled |
|  |  | Spatial | Ambient Versus Focal Percentage |  |  | Kinematic | Hand Average Acceleration |
|  |  |  | Number of Horizontal Saccades |  |  |  | Hand Average Velocity |
|  |  |  | Large Saccades |  |  |  | Hand Number of Stops |
|  |  |  | Number of Fixations |  |  |  | Hand Percentage of Short Stops |
|  |  |  | Number of Saccades |  |  |  | Hand Standard Deviation of Acceleration |
|  |  |  | Small Saccades |  |  |  | Hand Standard Deviation of Velocity |
|  |  |  | Total Distance Travelled |  |  |  | Head Average Acceleration |
|  |  |  | Total Length of Horizontal Saccades |  |  |  | Head Average Velocity |
|  |  |  | Total Length of Vertical Saccades |  |  |  | Head Number of Stops |
|  |  |  | Number of Vertical Saccades |  |  |  | Head Percentage of Short Stops |
|  |  | Kinematic | Average Acceleration |  |  |  | Head Standard Deviation of Acceleration |
|  |  |  | Average Velocity |  |  |  | Head Standard Deviation of Velocity |
|  |  |  | Standard Deviation of Acceleration |  |  | Interaction | Number of Object Bought |
|  |  |  | Standard Deviation of Velocity |  |  |  | Number of Objects Picked and Dropped |
|  | Zonal | Temporal | Time of First Visit of AOIx |  |  |  | Total Purchased Price |
|  |  |  | Total Time of AOIx |  | Zonal | Temporal | Hand Total Time of AOIx |
|  |  | Spatial | Number of Visits of AOIx |  |  |  | Hand Time of First Visit of AOIx |
|  |  |  | Total Distance Travelled in AOIx |  |  |  | Head Total Time of AOIx |
|  |  | Kinematic | Average Acceleration in AOIx |  |  |  | Head Time of First Visit of AOIx |
|  |  |  | Average Velocity in AOIx |  |  | Spatial | Hand Number of Visits of AOIx |
|  |  |  | Standard Deviation of Acceleration in AOIx |  |  |  | Hand Total Distance Travelled in AOIx |
|  |  |  | Standard Deviation of Velocity in AOIx |  |  |  | Head Number of Visits of AOIx |
| Navigation | General | Temporal | Average Duration of Stops |  |  |  | Head Total Distance Travelled in AOIx |
|  |  |  | Total Time |  |  | Kinematic | Hand Average Acceleration in AOIx |
|  |  | Spatial | Total Distance Travelled |  |  |  | Hand Average Velocity in AOIx |
|  |  | Kinematic | Average Acceleration |  |  |  | Hand Standard Deviation of Acceleration in AOIx |
|  |  |  | Average Velocity |  |  |  | Hand Standard Deviation of Velocity in AOIx |
|  |  |  | Number of Stops |  |  |  | Head Average Acceleration in AOIx |
|  |  |  | Percentage of Short Stops |  |  |  | Head Average Velocity in AOIx |
|  |  |  | Standard Deviation of Acceleration |  |  |  | Head Standard Deviation of Acceleration in AOIx |
|  |  |  | Standard Deviation of Velocity |  |  |  | Head Standard Deviation of Velocity in AOIx |
|  | Zonal | Temporal | Time of First Visit of ZOIx | Notes:  AOIx in Eye-tracking: Shelf_Down, Shelf_Middle, Shelf_Up, Adjacent, Near, Far  AOIx in Posture & Interaction/Hand: Shelf_Down, Shelf_Middle, Shelf_Up, Adjacent_Down, Adjacent_Middle, Adjacent_Up, Near_Down, Near_Middle, Near_Up, Far  AOIx in Posture & Interaction/Head: Adjacent_Down, Adjacent_Middle, Adjacent_Up, Near_Down, Near_Middle, Near_Up, Far  ZOIx in Navigation: Adjacent, Near, Far | | | |
|  |  |  | Total Time of ZOIx |  |  |  |  |
|  |  | Spatial | Number of Visits of ZOIx |  |  |  |  |
|  |  |  | Total Distance Travelled in ZOIx |  |  |  |  |
|  |  | Kinematic | Average Acceleration in ZOIx |  |  |  |  |
|  |  |  | Average Velocity in ZOIx |  |  |  |  |
|  |  |  | Standard Deviation of Acceleration in ZOIx |  |  |  |  |
|  |  |  | Standard Deviation of Velocity in ZOIx |  |  |  |  |

# Appendix B

Table B 1 Factor analysis of BFI-2-S

| **Questions** | **Negative Emotionality** | **Extraversion** | **Open Mindedness** | **Conscientiousness** | **Agreeableness** |
| --- | --- | --- | --- | --- | --- |
| **4** | 0.321302727 | -0.125810984 | -0.056294204 | 0.105797574 | 0.241791757 |
| **9** | 0.608092262 | -0.328984336 | 0.209352481 | -0.219878441 | 0.195713918 |
| **14R** | -0.779831567 | 0.177995475 | 0.010412869 | 0.059276922 | -0.072813282 |
| **19R** | -0.76339175 | -0.131547456 | 0.057547023 | 0.107173417 | -0.283809276 |
| **24R** | -0.530124985 | 0.476954138 | -0.119244661 | 0.223222517 | -0.299059842 |
| **29** | 0.635296675 | 0.032414019 | 0.018664139 | 0.21931845 | 0.006999 |
| **1R** | 0.046532577 | -0.503674449 | 0.211425791 | 0.357962257 | 0.062753868 |
| **6** | -0.016867152 | 0.385323044 | 0.251978575 | 0.075749976 | 0.012328848 |
| **11** | -0.156951622 | 0.620308099 | 0.174614408 | 0.172503724 | 0.11300451 |
| **16** | 0.066014368 | 0.730663736 | 0.09817232 | -0.134111137 | -0.068600833 |
| **21R** | 0.075700173 | -0.300508243 | -0.204511494 | -0.03485361 | 0.115473398 |
| **26R** | 0.169263381 | -0.572968346 | 0.166936497 | -0.079498393 | 0.234221063 |
| **5** | 0.213977681 | -0.051483435 | 0.604978363 | 0.0768733 | 0.035782252 |
| **10R** | 0.140569413 | -0.076096636 | -0.650247058 | 0.127501429 | -0.074636196 |
| **15** | -0.110344688 | 0.388434296 | 0.677371377 | -0.104270507 | -0.37704702 |
| **20R** | -0.128564314 | 0.037875286 | -0.588249398 | 0.067835066 | -0.021081968 |
| **25** | -0.061886007 | 0.11417293 | 0.171169288 | 0.021478833 | -0.312010743 |
| **30R** | 0.127788805 | -0.059970917 | -0.447015885 | 0.062910407 | 0.335120158 |
| **3R** | 0.045820843 | 0.268903298 | 0.325946058 | -0.372038484 | -0.004616861 |
| **8R** | 0.26663314 | -0.050411102 | 0.13238964 | -0.368321606 | 0.496363689 |
| **13** | -0.029647202 | 0.427459904 | 0.392133582 | 0.347446931 | 0.019103126 |
| **18** | -0.106917732 | 0.137606845 | -0.173514019 | 0.668798101 | -0.105278823 |
| **23** | -0.087212641 | 0.320370633 | 0.058223764 | 0.771489383 | 0.042949686 |
| **28R** | 0.045015691 | 0.170429844 | -0.164303139 | -0.571392053 | 0.430317907 |
| **2** | -0.039637759 | 0.0283497 | 0.102037778 | -0.062609019 | 0.492486336 |
| **7R** | 0.408586452 | 0.060111685 | -0.028985915 | -0.129283408 | -0.185268499 |
| **12** | 0.062138874 | 0.44466637 | -0.001141415 | 0.228633798 | 0.28453562 |
| **17R** | 0.290199504 | -0.216104972 | 0.043960601 | -0.128818824 | -0.13908267 |
| **22** | -0.297970285 | 0.23924485 | 0.632690564 | 0.372246252 | 0.402038941 |
| **27R** | 0.381456129 | 0.016181915 | -0.306333893 | -0.175420611 | -0.021703711 |

Table B 2 Aggregated correlation of BFI-2-S questions within each domain

|  | **Negative Emotionality** | **Extraversion** | **Open Mindedness** | **Conscientiousness** | **Agreeableness** |
| --- | --- | --- | --- | --- | --- |
| **Negative Emotionality questions** | 0.60634 | 0.212284 | 0.078586 | 0.155778 | 0.183365 |
| **Extraversion questions** | 0.088555 | 0.518908 | 0.184607 | 0.142447 | 0.101064 |
| **Open mindedness questions** | 0.130522 | 0.121339 | 0.523172 | 0.076812 | 0.192613 |
| **Conscientiousness questions** | 0.096875 | 0.229197 | 0.207752 | 0.516581 | 0.183105 |
| **Agreeableness questions** | 0.246665 | 0.167443 | 0.185858 | 0.182835 | 0.254186 |

Table B 3 Factor loadings and cumulative variance of the BFI-2-S questionnaire

|  | **Factor 1** | **Factor 2** | **Factor 3** | **Factor 4** | **Factor 5** |
| --- | --- | --- | --- | --- | --- |
| **SS Loadings** | 3.114805 | 3.032921 | 2.951164 | 2.42629 | 1.705121 |
| **Proportion variance** | 0.103827 | 0.101097 | 0.098372 | 0.080876 | 0.056837 |
| **Cumulative variance** | 0.103827 | 0.204924 | 0.303296 | 0.384173 | 0.44101 |
